# Antenatal depression and associated factors among pregnant women attending antenatal care at public health facilities in the Gida Ayana district, Oromia Region, West Ethiopia, in 2022

**DOI:** 10.3389/fpubh.2023.1176703

**Published:** 2023-10-09

**Authors:** Lelisa Oljira, Eba Abdissa, Matiyos Lema, Emiru Merdassa, Jira Wakoya Feyisa, Markos Desalegn

**Affiliations:** ^1^Department of Public Health, Institute of Health Sciences, Wollega University, Nekemte, Ethiopia; ^2^Department of Psychiatry Nursing, School of Nursing, Institute of Health Sciences, Wollega University, Nekemte, Ethiopia

**Keywords:** perinatal, antenatal, depression, women, antenatal care, pregnant

## Abstract

**Background:**

Though antenatal depression (AND) has a risk of maternal and fetal morbidity and mortality, it is a neglected component of pregnancy care in Ethiopia. Research evidence is compulsory in different parts of the country to alleviate this problem. Thus, this study was needed to assess antenatal depression and its associated factors, which can help antenatal care (ANC) providers and program coordinators focus on the mental health of pregnant mothers.

**Objectives:**

This study aimed to assess antenatal depression and associated factors among pregnant women attending ANC at public health facilities in the Gida Ayana district, Oromia Region, West Ethiopia, in 2022.

**Methods:**

A facility-based cross-sectional study was conducted among 370 pregnant women attending ANC at public health facilities. Systematic random sampling techniques were used to select study participants. A standard (validated) tool, the Edinburgh Postnatal Depression Scale, was also used to assess antenatal depression. The collected data were coded, entered into Epi-data software version 4.6, and analyzed by SPSS version 23. Multivariable logistic regression analyses were used to identify associated factors with a *p*-value <0.05.

**Results:**

In this study, the prevalence of antenatal depression was 62 (16.8%; 95% CI: 13, 20.5). Being single in marital status (AOR = 3, 95% CI: 1.5, 6.2), having an unplanned pregnancy (AOR = 2.7, 95% CI: 1.45, 5.1), and having partner conflict (AOR = 3.49, 95% CI: 1.79, 6.8) were the factors associated with antenatal depression.

**Conclusion:**

About one in five pregnant women has antenatal depression. Being single, having an unplanned pregnancy, and having a dissatisfied relationship with a sexual partner were the factors associated with antenatal depression. Therefore, women or partners are expected to plan pregnancy, and the dissemination of health information related to an unplanned pregnancy needs to be intensified by health providers. The partner ought to avoid conflict during the pregnancy, and healthcare providers or families are needed to support the single or widowed pregnant women. Further prospective cohort studies are needed to ascertain the effect of antenatal depression on fetal–maternal outcomes.

## 1. Background

Prenatal depression is a circumstance that labels a range of physical and emotional changes encountered by women during pregnancy (1, 2). Some of these symptoms include fatigue, crying, hopelessness, anxiety, changes in appetite, sleeping difficulty, a lack of interest in daily activities, and suicide (1, 2). The mental health of women in the reproductive age group is becoming a significant public health problem both in developing and developed countries (3).

Globally, ~280 million people suffer from depression annually (4). The prevalence of antenatal depression ranges from 15 to 65% around the globe (5). According to a study conducted in one of the sub-Saharan countries, Ghana, the prevalence of antenatal depression was 19.7%, while 14.1% of them were confirmed to have suicidal ideation (6). Evidence showed that the prevalence of prenatal depression in Ethiopia was 24.2% until 2018 (7). In the West Shawa zone of Oromia, the prevalence of prenatal depression was 32.3% (8).

Antenatal depression is the leading cause of disability, poor performance, suicide, premature death, discrimination, and stigma among pregnant women it affects (4, 9). It also has negative effects on the fetus, including low birth weight and preterm birth, which carry a great risk of neonatal death (10). Furthermore, antenatal depression is a confirmed risk factor for stunting in a child born from a pregnancy suffering from antenatal depression (11). In addition, a surviving child born to a woman affected by antenatal depression has a high risk of developing lifelong mental impairments (12). Moreover, antenatal depression is associated with severe pregnancy complications, including preeclampsia, a high rate of cesarean delivery, birth asphyxia, and postpartum psychosis, among others (6, 13, 14). Despite its severity and seriousness, it was the most neglected element of antenatal care (13–17).

The factors associated with antenatal depression include lack of basic needs and safety, low-income level, poor social status, unplanned pregnancy, spousal violence, sexual harassment, personal and family history of mental disorder, substance abuse, marital status, being HIV positive, poor social support, bad obstetric history, fear of labor pain, and lack of ANC, among others (4–17). Even though most of the identified risk factors for antenatal depression are believed to be preventable by appropriate, timely action, failure to recognize them and delays in seeking healthcare result in further expenses to treat the complications that result every year (5).

In Ethiopia, the Program for Improving Mental Health Care (PRIME) and the Emerging Mental Health System in Low- and Middle-Income Countries (Emerald) have been working to improve maternal mental health (18, 19). The program for improving mental healthcare aimed to scale up mental health service integration through evidence generation (18), whereas the Emerging Mental Health System in Low- and Middle-Income Countries (Emerald) aimed to improve the effectiveness of mental health service delivery by identifying health system obstacles and their solutions (19).

Despite this, antenatal depression is still a critical public health problem due to its intergenerational impact on mothers, infants, and children (20). One-third to one-fifth of women in developing countries have a significant mental health problem during pregnancy and after delivery (21, 22). Overall, the prevalence of antenatal depression varies among previous studies conducted in different parts of the world and different areas of Ethiopia. Furthermore, the factors reported as associated with antenatal depression among different studies were mostly not consistent with each other.

To the best of the knowledge of the investigator, the prevalence of antenatal depression and the factors associated with it were not studied in the East Wollega Zone, especially in the Gida Ayana district. There is no guideline to assess and treat AND in Ethiopia, and it is a neglected component of antenatal care. More importantly, since antenatal depression is a neglected component of antenatal care and leads to tremendous health, social, and economic negative consequences, adding more research evidence on the issue is necessary to arouse the focus of policymakers to solve the problem. Thus, this study was aimed at filling the existing gap.

## 2. Methods

### 2.1. Study area, period, and design

A facility-based cross-sectional study design was conducted from 1 March to 10 April 2022. The study was conducted in the Gida Ayana District, East Wollega Zone, Oromia region, west Ethiopia. The total population of the district was 158,635 people, of whom 79,878 were men and 78,757 were women. The district has 29 kebeles and 29,644 households. The district has 22 health posts, six public health centers, one public general hospital, and 26 private clinics (23).

### 2.2. Participants

All pregnant women in the Gida Ayana District were the source population, whereas pregnant women attending antenatal care at selected public health facilities in Gida Ayana District during the study period were the study population. All pregnant women attending antenatal care at the public health facilities of Gida Ayana district during this study period were included, whereas pregnant women who had a severe illness and those unable to communicate verbally were excluded from the study.

### 2.3. Sample size determinations

A single population proportion formula was used to determine the sample size for the prevalence of AND. The assumptions were as follows: a 95% confidence interval, a 5% margin of error, a 32.3% expected proportion of antenatal depression from a previous study done in the West Shawa Zone (8), and a 10% non-response rate were considered. Here, *n*_°_ = sample size; ρ = proportion from the previous study; *d* = desired degree of precision; *Z* = level of confidence desired, at a 95% confidence level.


$$\begin{array}{l} n_{{}^{\circ}}=\frac{{\left(Z\alpha /2\right)}^{2}\times \rho \left(1-\rho \;\right)}{d^{2}} \end{array}$$

The double population proportion formula was employed to estimate the sample size for factors associated with antenatal depression using EPI INFO version 7. Considering previous abortion as an associated factor in prenatal depression, the power of the study was 80% (95% CI), the ratio of unexposed to non-exposed was 1, the proportion among exposed was 47.8%, and the proportion among non-exposed was 27.3% (24). This assumption yields a sample size of 215, which is less than the one estimated for the prevalence of antenatal depression. Therefore, the sample size calculated for the prevalence of AND was used as the maximum sample size of 370 after adding a 10% non-response rate.

### 2.4. Sampling technique

A systematic random sampling method was employed to pick the study participants. The district has six public health centers and one public general hospital, and all of them were included in the study. Based on the record of the last 6 months, the average number of antenatal mothers expected to attend all the public health facilities during the data collection period was identified. Then the sample was proportionally allocated to each health facility based on their respective client flow. The individual client was approached by calculating the sampling interval “*k* = *N*/*n*,” where “*N*” is the total number of mothers expected to attend ANC at each health facility during the data collection period and “*n*” is the proportionally allocated sample size for each health facility. Thus, the sampling interval “*k*” becomes 1.4, which was rounded to 2 for all health facilities [Ayana Health Center (67/49), Angara Gute Health Center (74/54), Jangir Health Center (42/31), Gaba Jimata Health Center (35/24), Andode Dicho Health Center (42/31), Warabo Health Center (44/32), and Gida Ayana General Hospital (205/149)]. The first respondent was selected randomly, and the subsequent respondents were selected from every other mother visiting the health facility for ANC.

### 2.5. Data collection tools and procedures

A face-to-face interviewer-administered structured questionnaire was used to collect the data. The questionnaire has five parts: socio-demographic characteristics, obstetric characteristics, predisposing factors (behavioral pattern and history of depression), social support, domestic violence, and antenatal depression measurements were adapted (15, 16, 20). The tool was prepared in English and translated into Afaan Oromo, and back again to English by different professionals in both Afaan Oromo and English to maintain uniformity. The Edinburgh Postnatal Depression Scale (EPDS), a 10-item self-reporting questionnaire that has been validated for screening antenatal depression, was also used to assess antenatal depression (AND) (25).

Two experienced female midwives and two supervisors participated in data collection for 5 weeks. The supervisors led the data collectors during the data collection procedure. Ahead of data collection, both the supervisors and data collectors were given training on the objective of the study, the content of the tool, and an approach to respondents during data collection. Supervisors checked the accuracy and completeness of the data and submitted it to the principal investigator. The principal investigator oversaw the whole data collection process.

### 2.6. Data quality control

Training the supervisors and female data collectors was performed to ensure the quality of the data. A pilot test was done ahead of actual data collection among 5% of the sample size in the Guto Gida district of Uke, the health center, which has almost the same socio-demographic as the population of the study area. The amendment was made to the questionnaire based on the results of the pilot test. During and before data collection, the data collected by the data collectors have been checked for completeness and truthfulness daily by immediate supervisors.

### 2.7. Study variables

Dependent variable: antenatal depression among women attending ANC.

Independent variables: socio-demographic variables: age of the mother, maternal educational level, residence, family income, marital status, occupational status, ethnicity, and religion.

Obstetric-related variables: history of spontaneous abortion, stillbirth, congenital malformation, gravity, parity, unplanned pregnancy, stressful life events during pregnancy, experienced death of the baby, HIV status, and undesired fetal sex.

History of depression: personal history of depression and family history of depression.

Social support status and domestic violence experience: marital conflict, conflict with husband during the current pregnancy, the nature of the relationship with the husband, intimate partner violence, and status of social support.

Behavioral pattern: husband and self-status regarding substance abuse (smoking cigar rate, chat chewing, and drinking alcohol).

### 2.8. Operational definitions

Antenatal depression: the scale consisted of 10 short statements. A mother checks off one of the four possible answers that are closest to how she has felt during the past week. Most mothers easily complete the scale in <5 min. Responses were scored 0, 1, 2, and 3 based on the seriousness of the symptom. Items 3, 5, and 10 were reverse scored (i.e., 3, 2, 1, and 0). The total score was found by adding together the scores for each of the 10 items. Then the respondents who had a sum score ≥13 were regarded as having antenatal depression, and the rest were regarded as not having antenatal depression (26).

Social support: social support is considered the support the pregnant woman gains from her husband, neighbor, and relevant others, such as relatives. It was measured by the single item that asks for the respondents' self-report of whether they are satisfied or not with the social support they gain when they need it (27).

Intimate partner violence is defined as a behavior by an intimate partner or ex-partner that causes physical, sexual, or psychological harm, including physical aggression, sexual coercion, psychological abuse, and controlling behaviors (28). Thus, it was measured by using a single item that asks for the respondents' self-report of whether they had any form of intimate partner violence to respond as either yes or no.

Substance abuse: it was measured by the participant's self-report (as “yes” or “no”) of whether they or their husband or sexual partner drinks alcohol, chews chat, and/or smokes cigars during their recent pregnancy without specifying the frequency by which they take the substances (5).

Relationship status: the respondents were asked, “Have you ever had any conflict with your partner during the current pregnancy?” with a response of “yes” or “no” in this study. Based on this, the respondent's response was dichotomized as “partner conflict” for a “yes” response and “no partner conflict” for a “no” response.

### 2.9. Data processing and analysis

The collected data were coded and cleaned to identify missing values, outliers, and inconsistencies. The coded data were checked for completeness, entered into EPI INFO, and exported to SPSS version 23 for statistical analysis. Descriptive statistics were presented using frequency and percentages in tables and figures. In bivariate analysis, candidate variables were identified with a *p*-value <0.25. In multivariable logistic regression, variables with a *p*-value of 0.05 were determined to be factors associated with antenatal depression. In addition, the odds ratio and 95% confidence interval were used to determine the strength of the association. Hosmer–Lemeshow goodness-of-fit test was used, and the result showed a *p*-value of >0.05, indicating that the model was a good fit. To investigate collinearity, a diagnostic test was run across all independent variables. The VIF was <10, indicating that there was no severe multi-collinearity among the independent variables.

## 3. Results

### 3.1. Socio-demographic characteristics of the respondents

All the study participants in this study were interviewed and responded to, with a response rate of 100%. The mean and standard deviation of the participant's age were 27.956.14, respectively, while the range was between 17 and 43. A significantly higher proportion (285, or 77%) of the study's participants was married. Furthermore, the majority of 336 (90.8%) of the respondents were from the Oromo ethnic group, followed by the Amhara ethnic group at 26 (7%; Table 1).

**Table 1:** Socio-demographic characteristics of women who were attending ANC visit in public health facilities of Gida Ayana district, West Ethiopia, 2022 (*n* = 370).

**Variables**	**Categories**	**Frequency**	**Percentage (%)**
Age	≤ 19	23	6.2
	20–29	211	57
	≥30	136	36.8
Marital status	Single	51	13.8
	Married	285	77
	Divorced	21	5.7
	Widowed	13	3.5
Religion	Protestant	184	49.7
	Orthodox	114	30.8
	Muslim	69	18.6
	Others^*^	3	0.9
Ethnicity	Oromo	336	90.8
	Amhara	26	7
	Others^**^	8	2.2
Residence	Rural	156	42.2
	Urban	214	57.8
Educational status	Able to read and write	77	20.8
	Unable to read and write	87	23.5
	Primary	48	13
	Secondary	93	25.1
	College/university	65	17.6
Occupational status	House wife	174	47
	Merchant	62	16.8
	Self-employed	53	14.3
	Government employee	81	21.9
Household monthly income	<2,500	180	48.6
	≥2,500	190	51.4

^*^Catholic, and Adventist.

^**^Tigre and Gurage.

### 3.2. Obstetric characteristics of the respondents

Approximately three-fourths (72.4%) of the respondents had two to five pregnancies or gravidity. In this study area, approximately a quarter (87, 23.5%) of the respondents had experienced spontaneous abortion, and the other 40 (10.8%) of the respondents had experienced stillbirth, whereas 47 (12.7%) of the respondents had experienced the death of their baby (Table 2).

**Table 2:** Obstetric characteristics of women who were attending ANC visit in public health facilities of Gida Ayana district, West Ethiopia, 2022 (*n* = 370).

**Variables**	**Categories**	**Frequency**	**Percentage (%)**
Gravidity	Primigravida	48	13
	2–5	268	72.4
	≥6	54	14.6
Parity	Nullipara	48	13
	2–5	307	83
	≥6	15	4
Current pregnancy status	Planned	189	51.1
	Not planned	181	48.9
Know their HIV status during the current pregnancy	Yes	270	73
	No	100	27
Attending their ANC as per appointment	Yes	154	41.6
	No	216	58.4
Had any complication during the current pregnancy	Yes	75	20.3
	No	295	79.7
Had fear of giving birth of the current pregnancy	Yes	96	25.9
	No	274	74.1
Had known the sex of their current fetus	Yes	39	10.5
	No	331	89.5
History of spontaneous abortion	Yes	87	23.5
	No	283	76.5

### 3.3. Behavioral patterns, social support status, and domestic violence experience among the respondents

Almost all the respondents had no history of smoking cigarettes (368, 99.5%), chewing chat (365, 98.6%), or drinking alcohol (355, 95.9%). In this study area, ~70 (18.9%) and 44 (11.9%) of pregnant women have experienced intimate partner violence (IPV) during their current pregnancy, respectively. Approximately one-fourth (23.2%) of the pregnant women in this study area had experienced marital conflict at least once in their marriage. Concerning their relationship status with their spouse or sexual partner, the majority (294, or 79.5) of them had a happy relationship. Approximately 250 (67.6%) of the participants had support from family or a partner during the current pregnancy.

### 3.4. Edinburgh Postnatal Depression Scale assessment of the respondents

Approximately 50% of the 181 (48.9) study participants laughed and saw the funny side of things all of the time in the last week. Fifty-three of the present 186 (50.30) study participants were anxious or worried for no good reason in the last week (Table 3). Based on this depression scale assessment, 62 (16.8%; 95% CI: 13, 20.5) of the study participants had antenatal depression.

**Table 3:** Edinburgh Postnatal Depression Scale assessment among women who were attending ANC visit in public health facilities of Gida Ayana district, West Ethiopia.

**Variables**	**Categories**	**Frequency**	**Percent (%)**
In the last week I have been able to laugh and see the funny side of things?	Yes, all of the time	181	48.9
	Yes, most of the time	150	40.5
	No, not very often	22	5.9
	No, not at all	17	4.6
In the last week I have looked forward with enjoyment to things	Yes, all of the time	149	52.4
	Yes, most of the time	133	35.9
	No, not very often	27	7.3
	No, not at all	16	4.3
In the last week I have blamed myself unnecessarily when things went wrong	Yes, most of the time	177	47.8
	Yes, some of the time	108	29.2
	Not very often	64	17.3
	No, neve	21	5.7
In the last week I have been anxious or worried for no good reason	Yes, all of the time	186	50.3
	Yes, most of the time	114	30.8
	No, not very often	48	13
	No, not at all	22	5.9
In the last week I have felt scared or panicky for no very good reason	Yes, most of the time	189	51.1
	Yes, some of the time	108	29.2
	Not very often	47	12.7
	No, neve	726	7
In the last week I have things been getting on top of me	Yes, most of the time	216	58.4
	Yes, some of the time	82	22.2
	Not very often	55	14.9
	No, neve	17	4.6
In the last week I have been so unhappy that I have had difficulty sleeping	Yes, most of the time	182	49.2
	Yes, some of the time	82	22.2
	Not very often	92	24.9
	No, neve	14	3.8
In the last week I have felt sad or miserable	Yes, most of the time	163	44.1
	Yes, some of the time	103	27.8
	Not very often	89	24.1
	No, neve	15	4.1
In the last week I have been so unhappy that I have been crying	Yes, most of the time	226	61.1
	Yes, some of the time	85	23
	Not very often	44	11.9
	No, neve	15	4.1
In the last week the thought of harming myself has occurred to me	Yes, most of the time	241	65.1
	Yes, some of the time	86	23.2
	Not very often	26	7
	No, neve	17	4.6

### 3.5. Factors associated with antenatal depression among the respondents

In bivariable analysis, age, marital status, residence, monthly income, having ever had a spontaneous abortion, fear of giving birth, or having had any complications recently, current status, pregnancy status, and status of relationship with a partner were found to be candidate variables for the multivariable logistic regression model.

Three variables were statistically significantly associated with the outcome variable in the final model of multivariable logistic regression. Thus, the variables that had a significant statistical association in the multivariable logistic regression analysis were marital status, whether the current pregnancy was planned, and the respondents' status of relationship with their sexual partner. The odds of having antenatal depression among single women were three times higher compared to married women (AOR = 3, 95% CI: 1.5, 6.2). Furthermore, the odds of having antenatal depression among the women who had unplanned pregnancies were 2.7 times higher compared to the women who had planned pregnancies (AOR =2.7, 95% CI: 1.45, 5.1). In addition, the odds of having antenatal depression among the women who had an unsatisfactory relationship with their sexual partner were 3.49 times higher compared to the women who had a satisfactory relationship with their sexual partner (AOR = 3.49, 95% CI: 1.79, 6.8; Table 4).

**Table 4:** Multivariable logistic regression analyses of factors associated with antenatal depression among pregnant women who were attending ANC in Gida Ayana district, West Ethiopia, 2022 (*n* = 370).

**Variables**	**Categories**	**Had antenatal depression**		**COR (95 % CI)**	**AOR (95%, CI)**

		**Yes (** ***N*** **, %)**	**No (** ***N*** **, %)**		
Age	≤ 19	7 (30.4)	16 (69.6)	1.85 (0.69, 4.96)	2.42 (0.75, 7.76)
	20–29	29 (13.7)	182 (86.3)	0.67 (0.38, 1.20)	0.7 (0.36, 1.38)
	≥30	26 (19.1)	110 (80.9)	1	1
Marital status	Single	17 (33.3)	34 (66.7)	3.15 (1.6, 6.18)	3 (1.5, 6.2)^*^
	Divorced/widowed	6 (17.6)	28 (82.4)	1.35 (0.53, 3.48)	0.46 (0.16, 1.3)
	Married	39 (13.7)	246 (86.3)	1	1
Ever had spontaneous abortion	Yes	20 (23)	67 (77)	0.584 (0.32, 1.06)	0.757 (0.39, 1.47)
	No	42 (14.8)	241 (85.2)	1	1
Had fear of giving birth	Yes	24 (25)	72 (75)	2.07 (1.16, 3.68)	1.75 (0.86, 3.58)
	No	38 (13.9)	236 (84.1)	1	1
Had any complication recently	Yes	18 (24)	57 (76)	0.55 (0.299, 1.03)	1.002 (0.46, 2.2)
	No	44 (24.9)	251 (85.1)	1	1
Current pregnancy status	Unplanned	44 (24.3)	137 (75.6)	3.05 (1.69, 5.5)	2.7 (1.45, 5.1)^*^
	Planned	18 (9.5)	171 (90.5)	1	1
Status of relationship with partner	Unsatisfactory	25 (32.9)	51 (67.1)	3.4 (1.9, 6.14)	3.49 (1.79, 6.8)^*^
	Satisfactory	37 (12.6)	2 57 (87.4)	1	1

^*^Indicates statistical significance at p-value < 0.05.

AOR, adjusted odds ratio; CI, confidence interval; COR, crudes odds ratio.

## 4. Discussion

Approximately one-fifth (16.8%) of the pregnant women attending ANC services at the public health facilities in Gida Ayana district had antenatal depression. Being single in marital status, having an unplanned pregnancy, and having a dissatisfied relationship with the sexual partner were identified as factors associated with antenatal depression in this study area.

A significant proportion of pregnant women in this study had antenatal depression, which was lower than the result of the study done in Addis Ababa, Ethiopia, where the prevalence of antenatal depression was 24.94% (26). In addition, the magnitude of antenatal depression was lower than that of the study conducted in West Shawa (32.3%) (8), Maichew, north Ethiopia (31.1%) (29), Badewacho, south Ethiopia (23.3%) (30), and Gondar Hospital, northwest Ethiopia (23%). The possible reason for the difference could be due to differences in the study period and differences in socio-economic or cultural characteristics among the study areas. On the other hand, the lower magnitude of antenatal depression in this area could also be due to less intimate partner violence (11.9%) during the current pregnancy, and the majority of the participants in this study area were multi-gravida (72.4%), which is related to a lower risk of antenatal depression.

The magnitude of antenatal depression in the current study was similar to the finding of the study done in Jimma town (16.6%) (1) and Dubti Hospital (17.9%) (14), and this similarity could be due to close geographical proximity and similar health system factors, and the cultures of communities within Ethiopia seem to complement each other. However, the other study from the northeast of Ethiopia, Northwest Debre Tabor Town, revealed a lower prevalence of antenatal depression of 11.8% (25) than the current finding. This could be because this study area was a conflict area where there could be catastrophic events, which could increase the risk of prenatal depression (31). Being single in marital status was associated with antenatal depression in the current study. This finding is consistent with the findings of the study done in Northwest Ethiopia and Southwest Ethiopia, where being single was associated with higher odds of antenatal depression (12, 30). The possible reason for the similarity could be that unmarried pregnant women lack the necessary support from their male partner and most probably face social stigma that leads them to develop antenatal depression (32).

Having an unplanned pregnancy was also positively associated with higher odds of antenatal depression in this study area. This finding was consistent with the findings of the studies (12, 17, 32). The women who had unplanned pregnancies are at risk of developing antenatal depression. The possible reason behind this is that it is obvious that if the pregnancy was not planned, the woman may be challenged to cope with any challenges that she may face during the pregnancy because “an unplanned pregnancy may have a long-lasting negative impact on a woman's perinatal mental health” (33).

Being in a dissatisfying relationship with a sexual partner was positively associated with higher odds of antenatal depression. This finding is in line with the findings of the study done in the Hadiya zone of south Ethiopia and Maichew, north Ethiopia (29, 30). The possible reason behind this could be that pregnant women with a dissatisfied relationship with their sexual partner are at increased risk of having antenatal depression (32). Evidence suggests that lower marital quality is highly associated with lower levels of life satisfaction and higher levels of depressive symptoms (33, 34).

The findings of this study are an input for healthcare providers and other relevant stakeholders at different levels to give due attention to preventing and treating antenatal depression during antenatal care visits. However, this study was not without limitations. First, this study was a facility-based study, which could miss pregnant women who were not attending antenatal care services at public facilities. On the other hand, the study used a cross-sectional study design, which cannot establish the temporal relationship between the independent variables and the outcome variable. Third, the study used a sole quantitative method that could not explore the concerns of pregnant women, spouses, family, the community, and the care provider about antenatal depression. In addition, contributing factors such as social support, intimate partner violence, substance abuse, and the relationship status of pregnant women could have been more comprehensively investigated through detailed inquiries or questionnaires.

## 5. Conclusion

Approximately one in five pregnant women in this study area had antenatal depression. Being single in marital status, having an unplanned pregnancy, and having a dissatisfied relationship with a sexual partner or spouse were possible factors associated with antenatal depression among pregnant women in this study area. Therefore, women and their partners ought to recognize that quality information, education, and family planning services can minimize the risk of unwanted pregnancy. Healthcare providers ought to intensify health information dissemination about the advantages of contraceptive methods in combating the risk of unplanned pregnancies. The spouses should avoid conflict during pregnancy. Single or unmarried pregnant women are better supported by family, community, and health professionals to minimize the actual or perceived social stigma. Orientation and in-service training should be provided for obstetric care providers regarding the detection, control, and prevention of antenatal depression to minimize further complications. Prospective cohort studies are needed to ascertain the effect of antenatal depression on fetal and maternal outcomes.

## Data availability statement

The raw data supporting the conclusions of this article will be made available by the authors, without undue reservation.

## Ethics statement

The Helsinki Declaration of Health Research ethical standard was applied to undertake this study (35). Ethical approval was obtained from the Institutional Review Board of Wollega University, Institute of Health. A support letter was obtained from the Gida Ayana district health office. Each health facility administrator was communicated with using the ethical approval letter and the support letter of their district. The study participants have been informed of the objective of the study, the right to discontinue or refuse to participate in the study, and the confidentiality of their responses. Written informed consent was obtained from each study participant before starting the data collection.

## Author contributions

LO and MD were involved in conducting the study, data analysis, report writing, and the main manuscript text development. EA, ML, EM, and JW were involved in editing the report and manuscript text development. Furthermore, they took part in preparing Tables 1–4. All authors reviewed the manuscript. All authors contributed to the article and approved the submitted version.
